# Prevalence and Prognostic Value of Mesenteric Artery Stenosis in Patients Undergoing Transcatheter Aortic Valve Implantation

**DOI:** 10.3389/fcvm.2022.750634

**Published:** 2022-02-07

**Authors:** Henri Lu, David Rotzinger, Pierre Monney, Olivier Muller, Michael Egea, Matthieu Grange, Eric Eeckhout, Matthias Kirsch, Salah D. Qanadli

**Affiliations:** ^1^Service of Cardiology, Lausanne University Hospital and University of Lausanne, Lausanne, Switzerland; ^2^Cardiothoracic and Vascular Unit, Department of Radiology, Lausanne University Hospital, Lausanne, Switzerland; ^3^Department of Medical Imaging, Neuchâtel Cantonal Hospital, Neuchâtel, Switzerland; ^4^Service of Cardiovascular Surgery, Lausanne University Hospital and University of Lausanne, Lausanne, Switzerland

**Keywords:** transcatheter aortic valve implantation, TAVI, TAVR, mesenteric artery stenosis, acute mesenteric ischemia

## Abstract

**Introduction:**

Data regarding the prevalence of mesenteric artery stenosis in patients undergoing transcatheter aortic valve implantation (TAVI) are scarce. Whether patients with high-risk features for acute mesenteric ischemia (AMesI) have a worse prognosis compared with those without high-risk features is unknown. We aimed to address these questions.

**Methods:**

We included 361 patients who underwent TAVI between 2015 and 2019. Using pre-TAVI computed tomography exams, the number of stenosed arteries in each patient and the degree of stenosis for the coeliac trunk (CTr), SMA and inferior mesenteric artery (IMA) were analyzed. High-risk features for AMesI were defined as the presence of ≥2 arteries presenting with ≥50% stenosis. Patient demographic and echocardiographic data were collected. Endpoints included 30-day all-cause mortality, mortality and morbidity related to mesenteric ischemia.

**Results:**

22.7% of patients had no arterial stenosis, while 59.3% had 1 or 2 stenosed arteries, and 18.0% presented stenoses in 3 arteries. Prevalence of significant stenosis (≥50%) in CTr, SMA, and IMA were respectively 11.9, 5.5, 10.8%. Twenty patients at high-risk for AMesI were identified: they had significantly higher all-cause mortality (15.0 vs. 1.2%, *p* < 0.001) and higher mortality related to AMesI (5.0 vs. 0.3%, *p* = 0.004), compared with non-high-risk patients.

**Conclusions:**

Patients at high-risk for AMesI presented with significantly higher 30-day all-cause mortality and mortality related to AMesI following TAVI. Mesenteric revascularization before TAVI interventions may be beneficial in these patients. Prospective studies are needed to clarify these questions.

## Introduction

Mesenteric artery stenosis (MAS) is a frequent incidental finding on abdominal imaging, with more than 90% of cases believed to be of atherosclerotic origin (1). The indications for revascularization in patients presenting with symptomatic MAS are well-established and codified (2), but the management of those with asymptomatic MAS is subject to debate. Although there is consensus that revascularization is not needed in patients with asymptomatic single-vessel stenosis, a decision not to intervene is less clear in those presenting with asymptomatic stenosis of 2 or more mesenteric arteries, as these patients may be at high risk for developing acute mesenteric ischemia (AMesI) (3). This is all the more important as the prevalence of asymptomatic MAS increases with age, being reported as 3% in patients under 65 years and up to 18% in those older than 65 years (4). Patients with aortic stenosis eligible for transcatheter aortic valve implantation (TAVI) are frequently diagnosed with asymptomatic MAS on routine pre-intervention aortic computed tomography (CT) imaging (5). The indication for mesenteric revascularization before TAVI procedures in this particular population is of special interest because TAVI by itself may induce transient hypotension and peripheral hypoperfusion via rapid ventricular pacing, thus theoretically increasing the risk of post-intervention digestive ischemia and digestive ischemia-reperfusion injury (6). To our knowledge, prognosis after TAVI of patients having MAS at baseline has never been studied. Therefore, we performed this single-center study using prospectively collected clinical and imaging data in patients undergoing TAVI to: (1) describe the prevalence and characteristics of asymptomatic MAS, (2) compare patients at high-risk for AMesI, vs. those not at high-risk, with regard to baseline clinical and echocardiographic characteristics, (3) compare the same patients with regard to 30-day all-cause mortality, mortality related to mesenteric ischemia, and incidence of AMesI.

## Methods

### Study Population

From January 1st 2015 to December 31st 2019, all patients who underwent TAVI interventions in our institution (Lausanne University Hospital, or CHUV), with available pre-intervention CT imaging data, were included. The CHUV serves as an academic tertiary-care hospital for a major part of the French-speaking population of Switzerland.

### Ethical Statement

All patients belonged to the SWISS TAVI Registry and provided written informed consent for the use of their data for research purposes. The study was conducted in accordance with the Declaration of Helsinki. Ethical approval was given by the local ethics commission (*Commission cantonale d'éthique de la recherche sur l'être humain*), decision CER-VD 211/13, dated May 10th, 2013.

### CT Image Acquisition and Analysis

CT imaging acquisitions were performed on a 256-row multidetector CT system (Revolution CT, GE Healthcare), with patients lying on their back, arms raised above their head, in a single breath-hold. Cardiac ECG-gated non-contrast images were first acquired to compute the aortic valve calcium score (these series were not analyzed as part of this study). In a second step, ECG-gated CT angiography of the carotid arteries, aorta, and iliac arteries was performed in the craniocaudal direction, following 100 mL of 350 mg/mL of iodinated contrast medium (Accupaque 350, GE Healthcare) injected into an antecubital vein (on the right side whenever possible). Images were analyzed by two radiologists, with any disagreement resolved by consensus or with the help of a third senior radiologist. Interrater variability for superior mesenteric artery (SMA) stenosis assessment has been evaluated previously (7). Data were prospectively collected in a dedicated file.

### Data Collection and Analysis

#### Imaging Data

**Figure 1:**
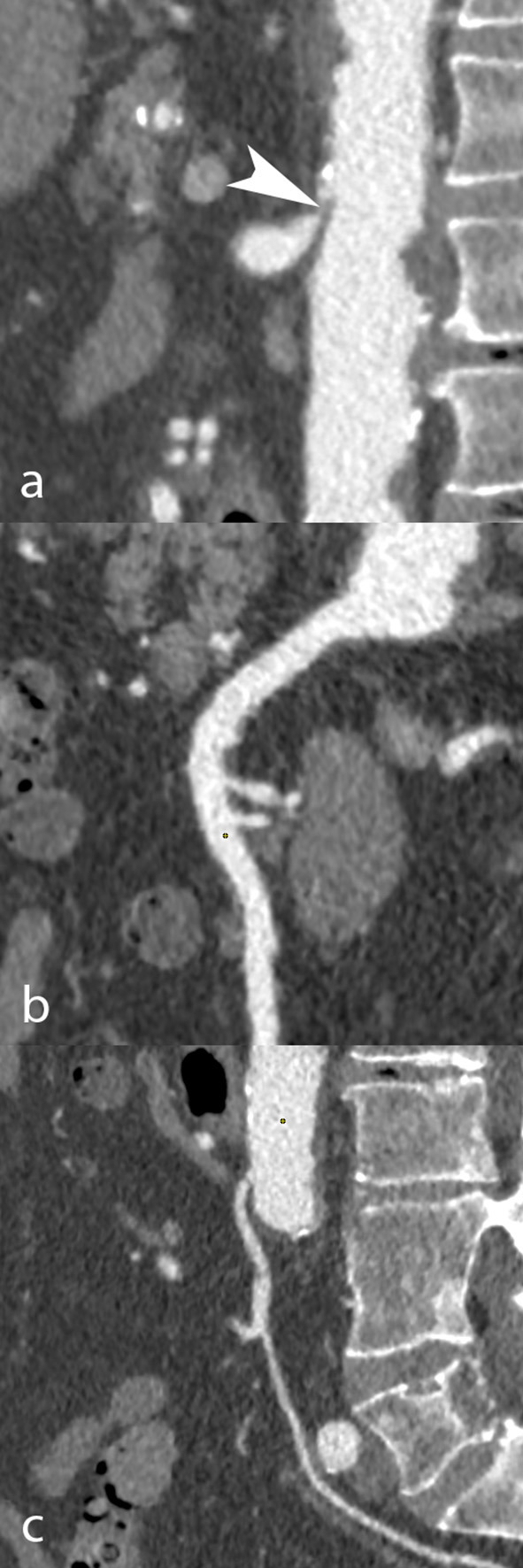
CT angiography in an 85-year-old man scheduled to undergo TAVI. Curvilinear reconstructions show the coeliac trunk **(a)**, superior **(b)**, and inferior mesenteric artery **(c)**. While the coeliac trunk was occluded due to the combination of an arcuate ligament and a mixed plaque (white arrowhead), the mesenteric arteries had no occlusive disease.

**Figure 2:**
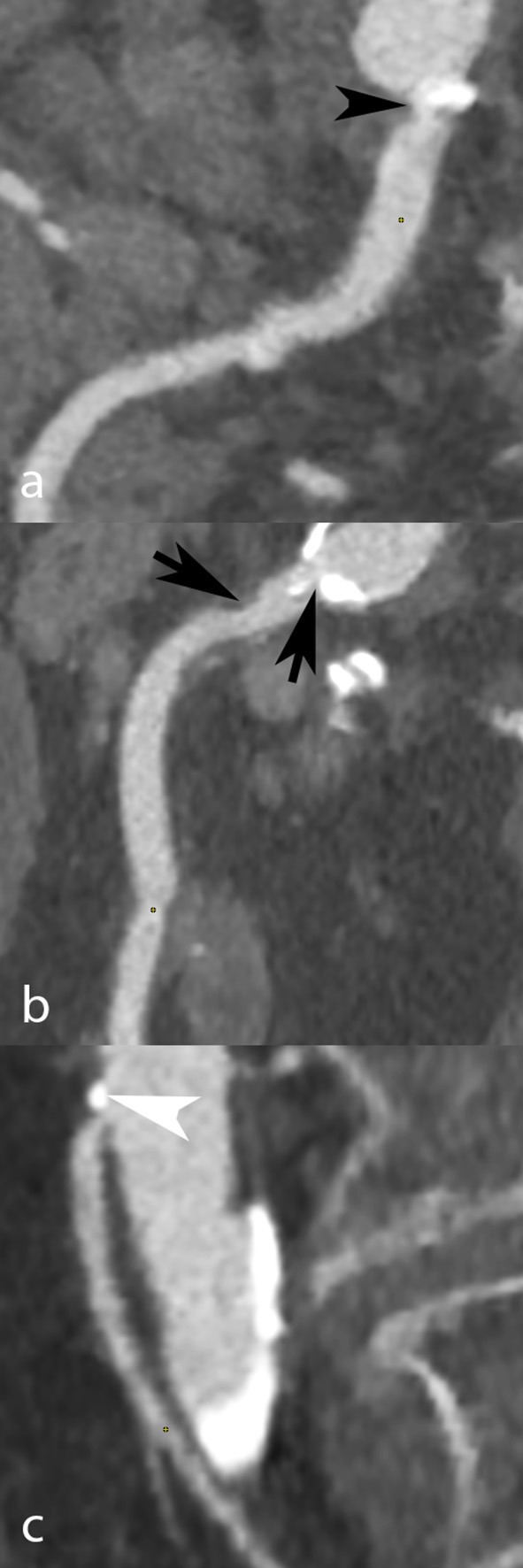
CT angiography in a 90-year-old woman scheduled to undergo TAVI. Curvilinear reconstructions show the coeliac trunk **(a)**, superior **(b)**, and inferior mesenteric artery **(c)**. All three arteries had significant ostial occlusive disease, due to mixed plaques at the coeliac (black arrowhead) and inferior mesenteric (white arrowhead) arteries' ostium, and to a mixed ostial plaque associated with a non-calcified post-ostial plaque (black arrows) in the superior mesenteric artery.

The coeliac trunk (CTr), superior mesenteric artery (SMA) and inferior mesenteric artery (IMA) were analyzed in all patients. Each artery was classified according to lumen narrowing expressed in percentage of the reference diameter: normal (0%), mild stenosis (<50%), moderate stenosis (50 to 69%), severe stenosis (70 to 99%), or occlusion (100%). The total number of stenosed arteries (0, 1, 2, or 3) in each patient, regardless of stenosis severity, was also collected. Patients at high-risk for AMesI were defined as those presenting with at least 2 arteries out of 3 presenting each with ≥50% stenosis (8). Figures 1, 2 show examples of CT-angiography with reconstructions of the CTr, SMA, and IMA, in patients undergoing pre-TAVI assessment.

#### Patient Data

Baseline patient demographic characteristics, including gender, age, body mass index (BMI), major comorbidities (such as diabetes mellitus and coronary artery disease), and echocardiographic data (left ventricular ejection fraction [LVEF]) were collected. Post-operative endpoints included 30-day all-cause mortality, 30-day mortality related to AMesI (as assessed by reviewing of medical files), all cases of AMesI within 30 days after TAVI. Only cases where the diagnosis of AMesI was confirmed based on an abdominal CT exam or perioperatively if the patient directly underwent abdominal surgery, were counted.

### Statistical Analysis

Categorical variables were expressed as frequencies and percentages and compared using Pearson's χ^2^ test. Continuous variables were reported as means with standard deviations (SDs) or medians with interquartile ranges (IQRs) and were tested for normality distribution using the Shapiro-Wilk test. Student's *t*-test was used to compare normally distributed continuous variables, whereas the Mann-Whitney test was used to compare non-normally distributed ones. Thirty-day survival curves were modeled using the Kaplan-Meier method and were analyzed using a log-rank test. Values with a *p* < 0.05 were considered statistically significant. The SPSS 27.0 software (SPSS Inc., Chicago, Illinois, USA) was used for all statistical analyses.

## Results

A total of 361 patients were included in our study. The distribution of the patients according to the number of stenosed arteries, regardless of stenosis severity, is presented in Table 1: 82 (22.7%) did not present any arterial stenosis, while 109 (30.2%) had 1 stenosed artery, 105 (29.1%) had 2 stenosed arteries, and 65 (18.0%) presented stenosis in all 3 arteries. Among the 361 patients, 20 at high-risk for AMesI were identified. Table 2 shows the repartition of patients by degree of stenosis for each artery. Overall, 197 patients (54.6%) presented with CTr stenosis of any degree, or occlusion, while 143 (39.6%) had SMA stenosis of any degree or occlusion, and 135 (37.4%) IMA stenosis of any degree or occlusion. Prevalence of moderate and severe stenosis associated with CTr, SMA, and IMA were, respectively 11.9, 5.5, 10.8%; the prevalence of occlusion was, respectively 0.5, 0.0, and 3.3%.

**Table 1:** Repartition of patients by number of stenosed arteries, regardless of stenosis severity.

**Number of stenosed arteries**	**Number of patients (%)**
0	82 (22.7)
1	109 (30.2)
2	105 (29.1)
3	65 (18.0)

**Table 2:** Repartition of patients by degree of stenosis for each artery.

**CT findings**	**Coeliac trunk**	**Superior mesenteric artery**	**Inferior mesenteric artery**
Normal	164 (45.4)	218 (60.4)	226 (62.6)
Mild (1–49%)	152 (42.2)	123 (34.1)	84 (23.3)
Moderate (50–69%)	35 (9.7)	19 (5.3)	35 (9.7)
Severe (70–99%)	8 (2.2)	1 (0.2)	4 (1.1)
Occlusion (100%)	2 (0.5)	0 (0.0)	12 (3.3)

*Values are expressed as n (%)*.

Baseline patient demographic, clinical, and echocardiographic characteristics are presented in Table 3. Compared with patients not at high-risk for AMesI, those at high-risk had significantly higher surgical risk, as assessed by the Euroscore II (5.31 [IQR, 3.86–16.45] vs. 3.87, [IQR, 2.23–6.42], *p* = 0.030) and higher prevalence of chronic obstructive pulmonary disease (35.0 vs. 16.1%, *p* = 0.030). No significant difference was found regarding the other baseline patient characteristics, although a trend toward lower BMIs and higher prevalence of dyslipidemia was found in high-risk patients.

**Table 3:** Baseline clinical and echocardiographic characteristics of patients at high-risk for mesenteric ischemia, vs. patients non at high-risk.

	**High-risk (*n* = 20)**	**Non-high risk (*n* = 341)**	***p*-value**
**Clinical characteristics**			
Age, years, median (IQR)	82.0 (76.0, 87.0)	83.0 (79.0, 87.0)	0.950
Male	7 (35.0)	159 (46.6)	0.311
BMI, kg/m^2^, median (IQR)	23.8 (20.8, 26.0)	25.6 (23.0, 29.7)	0.060
NYHA Functional class			
•I–II •III–IV	4 (20.0) 16 (80.0)	114 (33.4) 227 (66.6)	0.239
Euroscore II, median (IQR)	5.31 (3.86, 16.45)	3.87 (2.23, 6.42)	0.030
Chronic obstructive pulmonary disease	7 (35.0)	55 (16.1)	0.030
Diabetes mellitus	4 (20.0)	89 (26.1)	0.544
Dyslipidemia	15 (75.0)	182 (53.4)	0.059
Previous cardiac surgery	5 (25.0)	56 (16.4)	0.320
Coronary artery disease	12 (60.0)	171 (50.1)	0.392
Previous PCI	3 (15.0)	48 (14.1)	0.908
Hypertension	16 (80.0)	255 (74.8)	0.600
Stroke or TIA	4 (20.0)	47 (13.8)	0.438
Moderate to severe CKD	15 (75.0)	194 (56.9)	0.111
**Echocardiographic characteristics**			
LVEF			
•>50% •30–50% •<30%	15 (75.0) 4 (20.0) 2 (10.0)	245 (71.8) 73 (21.4) 22 (6.4)	0.775 0.876 0.536

*Patients were considered high-risk if they had at least 2 arteries out of 3 presenting each with ≥50% stenosis. Values are expressed as n (%), unless specified otherwise.TAVI, transcatheter aortic valve replacement; IQR, interquartile range; BMI, body mass index; PCI, percutaneous coronary intervention; NYHA, New York Heart Association; TIA, transient ischemic attack; CKD, chronic kidney disease; LVEF, left ventricle ejection fraction*.

### Post-operative Outcomes

**Figure 3:**
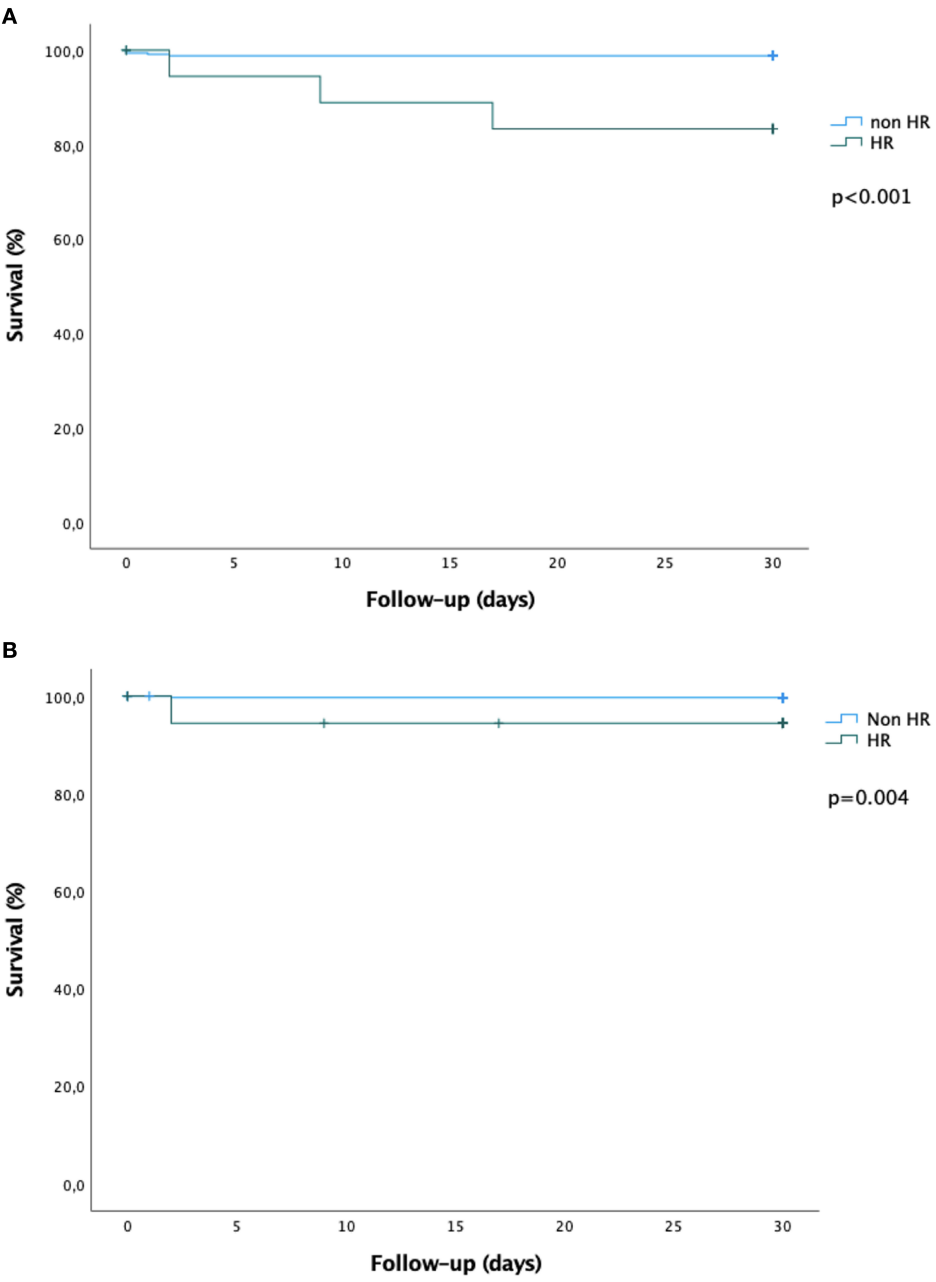
Kaplan-Meier survival curves at 30 days. **(A)** All-cause mortality. **(B)** Mortality related to digestive ischemia. The green curves represent the high-risk group, the blue curves represent the non-high-risk group. Survival curves were compared using the log-rank test.

Thirty-day outcomes are presented in Table 4. Patients at high-risk for AMesI had significantly higher all-cause mortality (15.0 vs. 1.2%, *p* < 0.001) and higher mortality related to AMesI (5.0 vs. 0.3%, *p* = 0.004). Among the three patients at high-risk for AMesI who died, one died of AMesI, while two died of extra-digestive causes (acute respiratory failure in both cases). There was a non-significant trend toward a higher incidence of AMesI in high-risk patients (5.0 vs. 0.9%, *p* = 0.065). Kaplan-Meier survival curves for 30-day all-cause mortality and mortality related to AMesI are presented in Figure 3.

**Table 4:** Postoperative endpoints of patients at high-risk for mesenteric ischemia, vs. patients non at high-risk.

	**High-risk (*n* = 20)**	**Non-high risk (*n* = 341)**	***p*-value**
All-cause 30-day mortality	3 (15.0)	4 (1.2)	<0.001
30-day mortality related to digestive ischemia	1 (5.0)	1 (0.3)	0.004
Digestive ischemia (all cases)	1 (5.0)	3 (0.9)	0.065

*Values are expressed as n (%)*.

## Discussion

Our results can be summarized as follows: (1) among the 361 patients undergoing pre-TAVI routine CT screening, 279 (77.3%) presented with at least one stenosed mesenteric artery and 20 (5.5%) were at high-risk for AMesI, (2) patients at high-risk for AMesI had significantly more comorbidities and higher surgical risk, compared with those not at high-risk, (3) patients at high-risk for AMesI at baseline presented with increased risks of 30-day all-cause mortality and 30-day mortality related to AMesI following TAVI.

Routine CT exams performed in patients eligible for TAVI constitute an important database to analyze asymptomatic MAS. This is all the more interesting as available data in the literature on asymptomatic MAS is very scarce. A previous retrospective study compared the prevalence of MAS in 73 patients planned for TAVI interventions who underwent routine CT imaging against 111 control patients who had CT imaging for other reasons (5). Stenoses with a lumen reduction of at least 50% were considered significant. 45.2% of TAVI patients had significant stenosis in at least 1 artery, and 8.2% of TAVI patients had significant stenosis in multiple arteries: these results are similar to our data. Interestingly, although the TAVI patients and control patients had no significant difference regarding demographic and clinical characteristics, the prevalence of MAS in control patients was lower (22.5% had at least 1 stenosed artery, and 1.8% had multiple stenosed arteries). This observation suggests that candidates for TAVI may present a higher atherosclerotic burden than the general population and is in agreement with previous reports (9, 10).

Although an increased risk of all-cause 30-day mortality was observed in patients at high-risk for AMesI compared with those not at high-risk, the two population groups were not comparable, as patients at high-risk for AMesI had a significantly higher surgical risk and more comorbidities. To which extent these differences regarding baseline characteristics have biased our results is unclear. Likewise, it is not possible to conclude if high-risk features for AMesI are mere markers of overall frailty or are independent predictors of mortality. In that sense, considering mortality related to AMesI may be more relevant, as potential confounding factors may less influence this endpoint. Concerning mortality related to AMesI in all our patients, it is worth noting that one previous study analyzing vascular complications in 102 patients undergoing TAVI found five major vascular complications, of which one was AMesI, with a fatal outcome at day 7. This rate of approximately 1% lies in the same range as our data (11).

One physio-pathological mechanism behind the increased mortality rate in patients at high-risk for AMesI may be the transient hypotension and peripheral hypoperfusion induced by rapid ventricular pacing. Briefly, the latter is necessary to implant balloon-expandable transcatheter heart valves (THVs) to reduce cardiac output and achieve cardiac standstill, thus allowing optimal positioning of the THVs (12). Rapid ventricular pacing may also be used with self-expandable THVs when pre- or post-dilatation of the aortic valve is needed. Patients at high-risk for AMesI at baseline may be more prone to developing overt digestive ischemia following rapid ventricular pacing. The same mechanism has been used to explain, among others, the pathogenesis of acute kidney injury after TAVI (13). Supporting this hypothesis, Fefer and colleagues, using a cohort of 412 patients undergoing TAVI, showed that patients who had three or more pacing episodes during TAVI procedures, compared with those who had no pacing, or 1 to2 pacing episodes, were significantly more likely to present prolonged procedural hypotension (respectively 25, 0, and 16%, *p* < 0.001) and suffered greater in-hospital mortality (6.5, 1.7, and 1.7%, *p* = 0.045) (6). From a physio-pathological standpoint, other factors that might precipitate overt AMesI in patients presenting high-risk features include embolic events, massive periprocedural bleeding, and anesthesia modality (local with sedation or general). However, these were not analyzed in our study.

The number of patients at high-risk for AMesI was relatively low (20 out of 361), yet the risk of 30-day mortality (all-cause and AMesI-related) was significantly increased. In our opinion, this highlights the importance of adequately screening and following these patients. The indication for pre-TAVI mesenteric revascularization should be tailored to each situation, and, in the absence of clear recommendations, it should be discussed on a case-by-case basis. This is all the more important as MAS is now easily treatable in most cases via an endovascular approach (percutaneous transluminal angioplasty and stenting). This approach has become the gold standard in most centers in the past decade and may be associated with significantly lower morbidity and mortality compared with open surgical revascularization (4, 14). After TAVI, the threshold to screen for AMesI should be particularly low in high-risk patients, especially since AMesI often presents with non-specific abdominal symptoms (postprandial abdominal pain, nausea/vomiting, diarrhea) (15). In all cases, cardiovascular secondary prevention measures are recommended to limit the progression of atherosclerotic disease (16).

## Limitations

Our study is subject to some limitations. First, patients at high-risk for AMesI and those not at high-risk for AMesI had significantly different baseline surgical risk and comorbidities; to which extent this may have affected the outcomes of interest is unclear. We tried to overcome this issue by adjusting for differences in comorbidities at baseline, but because of the small sample of patients with high-risk features and the relatively low overall mortality rate, statistical power was insufficient to consider a multivariable analysis. However, despite this limitation, our findings are hypothesis-generating. Secondly, we only compared 30-day outcomes after TAVI, but a more extended analysis period may be interesting. Finally, the results reported here are those of a single Swiss tertiary center and may not be transposable to other populations and centers.

## Conclusions

To our knowledge, this study is the first report evaluating the prognosis of patients at high-risk for AMesI after TAVI interventions. These patients presented with higher 30-day all-cause mortality and mortality related to AMesI, provided they had more comorbidities and higher surgical risk compared with those not at high-risk for AMesI. Although these results need to be confirmed with larger cohorts, they are hypothesis-generating in so far as mesenteric revascularization before TAVI interventions may be beneficial in patients presenting with high-risk features for AMesI. Further prospective studies are needed to clarify this question.

## Data Availability Statement

The raw data supporting the conclusions of this article will be made available by the authors, without undue reservation.

## Ethics Statement

The studies involving human participants were reviewed and approved by CER-VD 211/13. The patients/participants provided their written informed consent to participate in this study.

## Author Contributions

HL: data curation, formal analysis, methodology, and writing (original draft). DR, PM, and SQ: supervision, validation, and writing (review and editing). OM, ME, MG, EE, and MK: validation. All authors contributed to the article and approved the submitted version.

## Conflict of Interest

The authors declare that the research was conducted in the absence of any commercial or financial relationships that could be construed as a potential conflict of interest.

## Publisher's Note

All claims expressed in this article are solely those of the authors and do not necessarily represent those of their affiliated organizations, or those of the publisher, the editors and the reviewers. Any product that may be evaluated in this article, or claim that may be made by its manufacturer, is not guaranteed or endorsed by the publisher.
